# Relevance of in vitro agar based screens to characterize the anti-fungal activities of bacterial endophyte communities

**DOI:** 10.1186/s12866-016-0623-9

**Published:** 2016-01-16

**Authors:** Hanan R. Shehata, Eric M. Lyons, Katerina S. Jordan, Manish N. Raizada

**Affiliations:** Department of Plant Agriculture, University of Guelph, Guelph, ON N1G 2W1 Canada; Department of Microbiology, School of Pharmacy, Mansoura University, Mansoura, Egypt

**Keywords:** Endophyte, Plant, *Zea*, Biological control, Dual culture, *in planta*, Creeping bentgrass, *Sclerotinia homoeocarpa*, *Rhizoctonia solani*

## Abstract

**Background:**

Endophytes are microbes that inhabit internal plant tissues without causing disease. Plant microbial communities consist of large numbers of endophyte species. Understanding the functions of these endophytes is a major challenge. An important function of some endophytes is to suppress fungal pathogens. Typically, plant associated microbes are screened for anti-fungal activities in vitro using the high-throughput dual culture screen, but it is not clear whether this method correlates with the activities of these microbes *in planta*. Furthermore, it is not clear whether in vitro screening captures all of the microbes that show this activity inside plants. The objective of this study was to evaluate the relevance of the in vitro dual culture method for screening endophytes with anti-fungal activity.

**Results:**

In parallel, 190 bacterial endophytes from the corn grass family (*Zea*) were screened for suppression of two fungal pathogens (*Sclerotinia homoeocarpa* and *Rhizoctonia solani*) using the in vitro dual culture method, and *in planta* using the model plant, creeping bentgrass. All endophytes that showed anti-fungal activity *in planta* against *Sclerotinia homoeocarpa and Rhizoctonia solani *(3 or 4 strains, respectively, out of 190), were captured in vitro. The in vitro and *in planta* screening results strongly correlated (r = 0.81 and r = 0.94 for the two pathogens, respectively).

**Conclusions:**

Evidence was gained here that the in vitro dual culture method is a relevant method for high throughput screening of plant endophyte communities for anti-fungal activity. In our study, the method captured all of the microbes that suppressed the corresponding pathogens *in planta*.

**Electronic supplementary material:**

The online version of this article (doi:10.1186/s12866-016-0623-9) contains supplementary material, which is available to authorized users.

## Background

Plant microbiome communities consist of large numbers of species (endophytes) that can affect plant health, nutrition, growth and tolerance to abiotic and biotic stresses [1, 2]. A major function of plant associated microbes is to control plant pathogens, especially fungi [3]. This is usually mediated by direct antagonism through production of antimicrobials, competition for nutrients or space, or induction of host plant defenses [2]. Due to the complexity of plant microbiomes, a rapid method is necessary to screen for their beneficial activities to plants [3]. Obviously, screening hundreds of these microbes in whole plants is challenging [1, 4]. Researchers typically test for anti-fungal activities in vitro first, then carry forward only the positive candidates to plant-based assays, excluding the bulk of the microbes that showed no anti-pathogen activity [4–8]. Dual culture screens are widely used as they are high-throughput, but it is not clear if the results correlate with microbial activities *in planta* (i.e. whether microbes with *in planta* anti-fungal activities are missed). We could find few systematic comparisons of in vitro dual culture versus *in planta* screens for endophytes that suppress plant disease [9].

The objective of this study was to evaluate the relevance of the in vitro dual culture method as a systematic and comprehensive method for screening endophytes for anti-fungal activity. Here we screened 190 bacterial endophytes against fungal pathogens using *in planta* screens first then conducted in vitro dual culture screens. The endophytes were previously isolated from the seeds, shoots and roots of 14 genotypes of the corn family (genus *Zea*) [10, 11]. As *Zea* plants are large, we used a smaller genetic grass relative for *in planta* screens, namely creeping bentgrass (*Agrostis stolonifera* L.), as plants could grow and develop disease symptoms in tubes efficiently. Creeping bentgrass is one of the most widely used turfgrasses on golf courses [12]. Endophytes were screened for *in planta* suppression of two fungal pathogens, *Sclerotinia homoeocarpa* (dollar spot disease) [13] and *Rhizoctonia solani* (brown patch disease) [14]. *Sclerotinia homoeocarpa* is the most economically important disease that affects creeping bentgrass [13, 15] and is also widespread within the grass family [16]. *Rhizoctonia solani* is another major fungal pathogen that affects turfgrass species including creeping bentgrass [17]. *R. solani* affects diverse crops including potato, tomato, cucumber, green pea and rice [18–23].

## Results

### Screening for inhibition of *S. homoeocarpa*

**Fig. 1:**
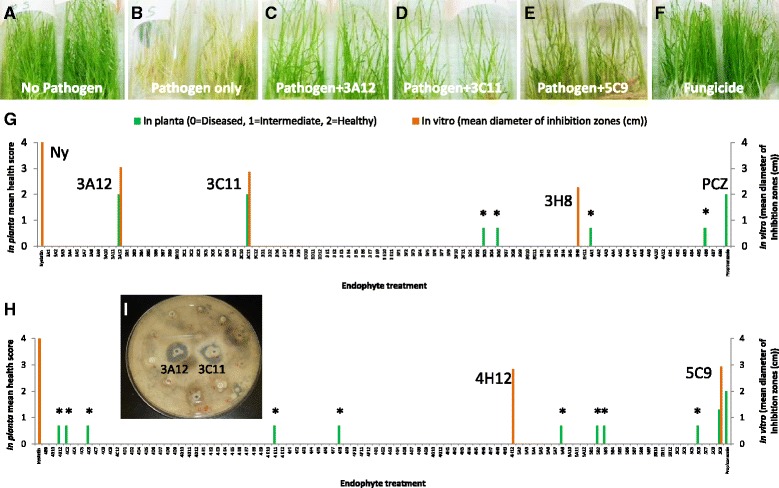
In vitro and *in planta* screening of maize endophytes for antifungal activity against *S. homoeocarpa*. **a**-**f**. *In planta* screen. Shown are tubes with creeping bentgrass treated with **a**. no fungal pathogen and no endophyte, **b**. the pathogen but no endophyte, **c**-**e**. the pathogen and successful anti-fungal endophytes, specifically **c**. endophyte 3A12, **d**. endophyte 3C11, and **e**. endophyte 5C9, **f**. fungicide treatment (Propiconazole). **g**-**h**. Graphs showing the results of in vitro and *in planta* screening for **g**. endophytes 1–95 (Additional file 1: Table S3) and **h**. endophytes 96–190 (Additional file 1: Table S3). The left y-axis is the plant visual health score per tube (average of 3 tubes, 30 plants per tube), based on the majority being very healthy (green, score of 2), very sick (showing chlorosis, score of 0) or intermediate (score of 1). An asterisk indicates that the *in planta* anti-fungal activity was not observed in Trial 2 (Additional file 2: Table S4). (I) An example PDA agar screening plate showing that *Zea* endophytes (3A12 and 3C11) create an inhibition zone of *S. homoeocarpa* growth in vitro. NY denotes Nystatin and PCZ denotes Propiconazole

*In planta* visual screening of 190 *Zea* endophytes for antifungal activity revealed that three endophytes (3A12, 3C11, 5C9) controlled *S. homoeocarpa* in creeping bentgrass in all 3/3 tubes tested (n = ~30 plants/tube) (Fig. 1a-e; Additional file 1: Table S3 and Additional file 2: Table S4). The antifungal strains were previously identified based on their 16S rRNA sequences (Additional file 3: Table S2 and Additional file 1: Table S3) [10, 11]. Strains 3A12, 3C11 and 5C9 were identified as different isolates of *Burkholderia gladioli*, isolated from diverse *Zea* genotypes (Additional file 2: Table S4). Interestingly, using the in vitro dual culture screen, the same endophytes plus two additions (3H8, 4H12) were found to form inhibition zones of *S. homoeocarpa* growth on agar in all three replicates (Fig. 1g-i; Additional file 1: Table S3 and Additional file 2: Table S4). Strains 3H8 and 4H12 were identified as *Bacillus subtilis* and *Paenibacillus polymyxa*, respectively (Additional file 1: Table S3) [10, 11].

### Screening for inhibition of *R. solani*

**Fig. 2:**
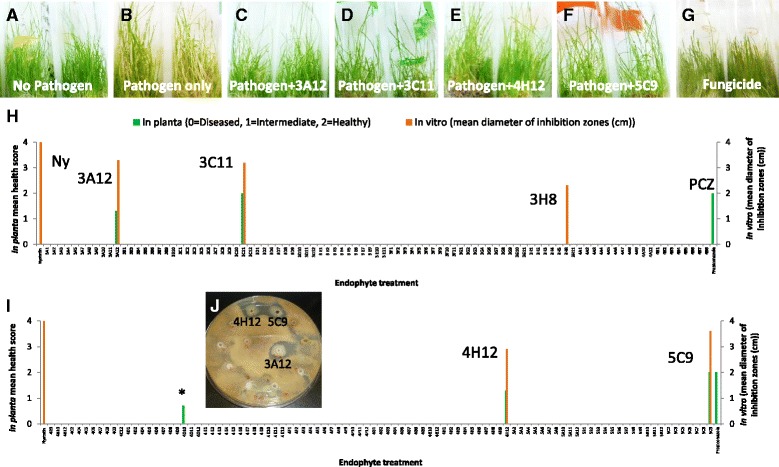
In vitro and *in planta* screening of maize endophytes for antifungal activity against *R. solani*. **a**-**g**. *In planta* screen. Shown are tubes with creeping bentgrass treated with **a**. no fungal pathogen and no endophyte, **b**. the pathogen but no endophyte, **c**-**f**. the pathogen and successful anti-fungal endophytes, specifically **c**. endophyte 3A12, **d**. endophyte 3C11, **e**. endophyte 4H12, and **f**. endophyte 5C9, **g**. fungicide treatment (Propiconazole). **h**-**i**. Graphs showing the results of in vitro and *in planta* screening for **h**. endophytes 1–95 (Additional file 1: Table S3) and **i**. endophytes 96–190 (Additional file 1: Table S3). The left y-axis is the plant visual health score per tube (average of 3 tubes, 30 plants per tube), based on the majority being very healthy (green, score of 2), very sick (showing chlorosis, score of 0) or intermediate (score of 1). An asterisk indicates that the *in planta* anti-fungal activity was not observed in Trial 2 (Additional file 2: Table S4). The right y-axis indicates the mean diameter of the zone of inhibition of *R. solani* on agar plates (n = 3). **j**. An example PDA agar screening plate showing that *Zea* endophytes (3A12, 4H12 and 5C9) create an inhibition zone of *R. solani* growth in vitro. NY denotes Nystatin and PCZ denotes Propiconazole

*In planta*, out of 190 endophytes screened, four endophytes (3A12, 3C11, 4H12, 5C9) controlled *R. solani* (Fig. 2a-f; Additional file 1: Table S3 and Additional file 2: Table S4) in all 3/3 tubes tested (n = ~30 plants/tube). Using the in vitro dual culture screen, the same endophytes, plus one addition (3H8) suppressed *R. solani* growth in all three replicates (Fig. 2h-i; Additional file 1: Table S3 and Additional file 2: Table S4). The taxonomic identities of these strains are noted above.

## Discussion

Parallel in vitro and *in planta* screens for anti-fungal activity have previously been reported, but with limited sample sizes. For example, amongst the largest parallel screens we could find, Aravind et al. [24] screened 74 endophytes from black pepper for activity against *Phytophthora capsici*. The authors identified 14 and 16–17 anti-fungal candidates based on in vitro and multiple *in planta* assays, respectively, but data was only presented for 12 strains that correlated [24]. Our study expands these results to 190 endophytes and two additional pathogens.

**Fig. 3:**
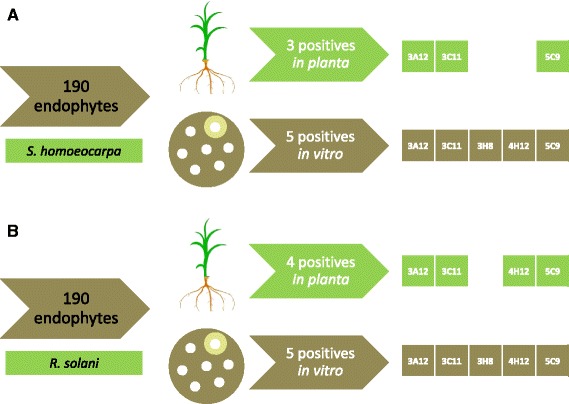
A cartoon illustrating the correlation between the in vitro and *in planta* screens for *Zea* endophytes that showed antifungal activity against both *S. homoeocarpa* and *R. solani*

Here, all three out of 190 endophytes that showed consistent anti-fungal activity against *S. homoeocarpa in planta*, were captured in vitro (Fig. 3a). Similarly, all 4 endophytes that showed consistent activity against *R. solani in planta* were captured in vitro (Fig. 3b). However, the in vitro screen identified additional endophytes with anti-fungal activities that did not show activity in our *in planta* screen. Taking into account all 190 endophytes initially tested, the results from in vitro and *in planta* screening of endophytes that combat *S. homoeocarpa* positively correlated (Pearson r = 0.61, *p* < 0.0001) (Additional file 1: Table S3). For the two anti-*R. solani* screens, the correlation was even greater (Pearson r = 0.89, *p* < 0.0001) (Additional file 1: Table S3). Excluding the *in planta* results that could not be replicated (in these cases, only 1 out of 3 tubes initially showed some healthy plants), the correlations were stronger (Pearson r = 0.80, *p* < 0.0001 for *S. homoeocarpa*; and r = 0.91, *p* < 0.0001 for *R. solani*) (Additional file 2: Table S4).

Our study shows that the in vitro screens captured all of the endophytes that showed anti-fungal activities *in planta*. These results may be interpreted in the context of the mechanisms by which endophytes control plant pathogens: induction of host resistance, direct antagonism, or competition [25, 26]. The in vitro screen had the capacity to detect endophytes that combat pathogens mainly through direct antagonism and possibly through competition for agar nutrients, but the screen could not have captured endophytes that induce host resistance or compete for ecological plant niches [4, 27–29]. Therefore it is surprising that the in vitro assays captured all the candidates identified from the *in planta* screens. There may be at least two hypotheses to explain these results:

First, the above defense mechanisms are not mutually exclusive. It was previously reported that some antifungal compounds can also stimulate host resistance [25]. Hence it may be that induction of host resistance is taking place in some of the anti-fungal candidates but it is coupled with direct antagonism, allowing the endophyte (s) to be identified by in vitro screening. Second, the endophytes here were screened in a host different from their native host which may have affected their potential to induce host resistance or to compete with the fungal pathogen for space. Consistent with this hypothesis, in the above study by Aravind et al., the authors used the native endophyte host to screen for antifungal activity and reported that at least 3/17 and 2/17 anti-fungal endophytes identified using two *in planta* screens were not apparently identified in vitro [24]. Here we may have been able to capture additional endophytes with anti-fungal activity *in planta* by testing other plant species susceptible to the same pathogens. However, in another *in planta* screen using annual ryegrass (*Lolium multiflorum*) with endophytes #1-75 (Additional file 1: Table S3), we have observed that the same two endophytes (3A12 and 3C11) suppress *S. homoeocarpa* (Additional file 4: Figure S1) as those that suppressed the pathogen in creeping bentgrass; these grasses belong to different genera. The third anti-fungal endophyte (5C9) also suppressed *S. homoeocarpa* in annual ryegrass (Additional file 1: Figure S1). In the future, it would be ideal to conduct parallel screens using the 14 native *Zea* hosts of these endophytes.

Our in vitro screens identified “false positives” with respect to the results *in planta*. This is consistent with previous studies. For example, Faltin et al. found that out of 17 plant associated microbes (including endophytes) that inhibited *R. solani* in vitro, 6 or 3 microbes, respectively, inhibited *R. solani* in lettuce leaf discs and in sugar beets [5]. In another study, 16/43 phylloplane and rhizosphere bacteria that inhibited *Phytophthora infestans* in vitro reduced disease symptoms by >10 % *in planta* in detached potato leaves with only two of these suppressing disease symptoms by >75 % [9]. Failure of some bacterial candidates from in vitro screens to suppress fungal disease when tested *in planta* may be attributed to many reasons, including: failure of endophytes to colonize the plant; failure of endophytes to migrate towards the pathogen; failure to compete with the native microbiota of their hosts; and/or sub-optimal production of anti-fungal compounds *in planta* due to pathways that compete for shared metabolic precursors, compared to optimized in vitro agar [4, 29]. Alternatively, as our *in planta* screen was visual, it may have missed endophytes that had only weak anti-fungal activities.

## Conclusions

There are practical and biological advantages and disadvantages to both in vitro and *in planta* screens for microbes with anti-fungal activity [29]. Nevertheless, our results and previous reports suggest that in vitro dual culture screening is a good method for high-throughput characterization of endophyte communities for anti-fungal activity, generally capturing the microbes that display these activities *in planta*.

## Methods

### Biological materials

Bacterial endophytes were isolated from seeds, roots and shoots of 14 different genotypes of *Zea* (Additional file 5: Table S1, Additional file 3: Table S2 and Additional file 1: Table S3) as previously reported [10, 11]. *Sclerotinia homoeocarpa* and *Rhizoctonia solani* were obtained from the Guelph Turfgrass Institute, Guelph, Canada. Creeping bentgrass (CB, *Agrostis stolonifera*, PENN A-4) seeds were obtained from the Ontario Seed Company, Kitchener, Canada. Annual ryegrass (AR, *Lolium multiflorum *L., Annuity) seeds were obtained from Seed Research of Oregon, USA.

### *In planta* screen

#### Preparation of endophyte coating agent mixture

Endophytes were cultured in LB overnight at 37 °C, shaking at 250 rpm. Cells were centrifuged, washed twice in 10 mM tris HCl (pH 7), then suspended to OD_595_ = 0.5. From each suspension, 500 μl were diluted in 5 ml of 9.3 % PVP aqueous solution (P-5288, Sigma, USA) and used to coat creeping bentgrass seeds.

#### Coating of seeds

CB seeds were surface sterilized by washing with 70 % ethanol for 1 min, bleach for 20 min, then rinsed 6 times with water. 100 CB seeds were added to each endophyte-PVP mixture (5 ml) and coated for 1 h on a rotary shaker.

#### Growing Turfgrass

Modified MS medium was used to germinate and grow CB, consisting of (per L, pH 5.8): half-strength modified basal salt MS (M571, Phytotech, USA), 250 μl nicotinic acid (1 mg/ml), 500 μl pyridoxine HCl (0.5 mg/ml), 5 ml thiamine HCl (100 mg/l), 500 μl glycine (2 mg/ml), 2 g Phytagel (P8169, Sigma, USA) in ddH_2_0. To solidify Phytagel, 0.166 g/l CaCl_2_ and 90 mg/l MgSO_4_ were added. Sterile MS (15 ml) was aliquoted into sterile 15 cm × 25 mm covered glass tubes (C5916, C5791, Sigma, USA). Per tube, 30 endophyte-coated CB seeds were placed on the media surface, in triplicate, allowed to germinate for 7 d in darkness, moved to a growth chamber (BTC-60, Enconair, Winnipeg, Canada) and grown under the following conditions: 25 °C constant, 16 h cool white fluorescent light (Philips F72T8/TL841/HO 65 W, 115–145 μmol m^−2^ s^−1^ measured using a Quantum BMQ Meter, Apogee Instruments, Logan, UT, USA).

#### Inoculation with pathogens

*S. homoeocarpa *and* R. solani* were grown on Potato Dextrose Agar (PDA) for 5 d at 28 °C. Discs from these plates were used to inoculate CB tubes after 10 d of plant growth. Controls were seeds coated with PVP but without endophytes, plus/minus each pathogen. For the fungicide treatment, Banner Maxx (Propiconazole 14.3 %, 60207-90-1, Syngenta Crop Protection, Canada) was used at a rate of 51 ml per 100 m^2^, applied as a spray 1 week after germination.

#### Re-inoculation of turfgrass

Two weeks after seed coating, glass tubes were re-inoculated with 100 μl of each endophyte cell suspension (OD_595_ = 0.5 in 10 mM tris HCl, pH 7). For control plants, 100 μl of 10 mM tris HCl (pH 7) were used.

#### Assessing disease symptoms *in planta*

Four weeks after germination, plants were assessed using a visual rating scale, based on the majority being very healthy (green, score of 2), very sick (showing chlorosis, score of 0) or intermediate (score of 1). Endophytes that showed anti-fungal activity were re-tested in an independent trial.

#### Testing candidate endophytes against *S. homoeocarpa *in annual ryegrass (*L. multiflorum*)

Annual ryegrass (AR) was used as another grass host to test the candidate antifungal endophytes against *S. homoeocarpa*. The same protocol for *in planta* screen was used with two modifications: 30 AR seeds were added to each endophyte-PVP mixture, and only 7 seeds were used per tube.

### In vitro screen

Dual culture screens were used. *S. homoeocarpa* and *R. solani* were cultured in YPD media at 25 °C, 80 rpm for 3 d. PDA was cooled to 50 °C, mixed with each fungal culture (1:25 v/v), then poured into Petri plates (150 mm × 15 mm). Holes were created in the agar using Pasteur pipettes; plugs were removed using a sterilized wire loop. In parallel, endophytes were cultured overnight in LB at 37 °C at 250 rpm, then OD_595_ adjusted to 0.4 to 0.6. Fifty microlitres of each culture were applied to the fungal plates in triplicate, incubated at 25 °C for 3–5 d, and then zones of inhibition of fungi were measured. Endophytes showing anti-fungal activity were re-tested in an independent trial. Nystatin (N581, PhytoTechnology Laboratories, USA) was used as a control fungicide at a concentration of 303 units/well.

### Statistical analysis

GraphPad Prism 6 was used for Pearson correlation analysis.

### Research involving plants

This study was conducted using local, commercially available plant seed, with plants grown entirely indoors followed by autoclaving. The study conforms to all Canadian guidelines, and does not require any special permissions or licenses.

### Availability of data and materials

The 16S rRNA sequences of endophytes used in this study have been deposited into Genbank (Additional file 3: Table S2). The authors agree to make available all microbial strains to non profit, public sector institutions.

## Additional files

Additional file 1: Table S3. List of all *Zea* endophytes used in this study and anti-fungal screening results. (XLSX 26 kb) Additional file 2: Table S4.
*In planta* re-testing of *Zea* endophytes that showed anti-fungal activity *in planta* in Trial 1. (XLSX 18 kb) Additional file 3: Table S2. Genebank accession numbers for 16S rRNA sequences of *Zea* endophytes used in this study. (DOCX 18 kb) Additional file 4: Figure S1.
*In planta* screening of maize endophytes for antifungal activity against *S. homoeocarpa* in annual ryegrass. Shown are tubes with annual ryegrass treated with (A) no fungal pathogen and no endophyte, (B) the pathogen but no endophyte, (C-E) the pathogen and successful anti-fungal endophytes, specifically (C) endophyte 3A12, (D) endophyte 3C11, and (E) endophyte 5C9, (F) fungicide treatment (Propiconazole). (PDF 198 kb) Additional file 5: Table S1. The origins of the *Zea* genotypes used to isolate the endophytes noted in this study. (DOCX 16 kb)

## Abbreviations

AR: annual ryegrass
CB: creeping bentgrass
PDA: potato dextrose agar
PVP: polyvinylpyrrolidone
YPD: yeast extract peptone dextrose
