# Supplementary material for: Relevance of in vitro agar based screens to characterize the anti-fungal activities of bacterial endophyte communities
Source: BMC Microbiol. 2016 Jan 16;16:8. doi: 10.1186/s12866-016-0623-9 (PMC4715354; doi:10.1186/s12866-016-0623-9)
Supplement: Additional file 3: Table S2. — Genebank accession numbers for 16S rRNA sequences of Zea endophytes used in this study. (DOCX 18 kb) [file 12866_2016_623_MOESM3_ESM.docx]

Additional file 3: Table S2. Genebank accession numbers for 16S rRNA sequences of *Zea* endophytes used in this study

| **ID** | **Genbank accession numbers** |
| --- | --- |
| 3A1 | JF753401 |
| 3A2 | JF753409 |
| 3A3 | JF753402 |
| 3A4 | JF753403 |
| 3A5 | JF753404 |
| 3A7 | JF753407 |
| 3A8 | JF753410 |
| 3A9 | JF753411 |
| 3A10 | JF753412 |
| 3A11 | JF753413 |
| 3A12 | JF753414 |
| 3B1 | JF753415 |
| 3B3 | JF753417 |
| 3B4 | JF753491 |
| 3B5 | JF753408 |
| 3B6 | JF753420 |
| 3B7 | JF753421 |
| 3B9 | JF753424 |
| 3B10 | JF753425 |
| 3C1 | JF753429 |
| 3C2 | JF753430 |
| 3C3 | JF753431 |
| 3C5 | JF753433 |
| 3C6 | JF753434 |
| 3C7 | JF753435 |
| 3C8 | JF753436 |
| 3C9 | JF753440 |
| 3C10 | JF753441 |
| 3C11 | KP455296 |
| 3C12 | JF753449 |
| 3D1 | JF753450 |
| 3D2 | JF753451 |
| 3D6 | JF753455 |
| 3D7 | JF753456 |
| 3D8 | JF753458 |
| 3D9 | JF753460 |
| 3D10 | JF753461 |
| 3D11 | JF753462 |
| 3D12 | JF753469 |
| 3 E1 | JF753463 |
| 3 E2 | JF753464 |
| 3 E3 | JF753465 |
| 3 E4 | JF753466 |
| 3 E5 | JF753467 |
| 3 E6 | JF753468 |
| 3 E7 | JF753470 |
| 3 E9 | JF753473 |
| 3 E10 | JF753475 |
| 3 E11 | JF753477 |
| 3F1 | JF753482 |
| 3F2 | JF753483 |
| 3F3 | JF753484 |
| 3F4 | JF753485 |
| 3F5 | JF753487 |
| 3F6 | JF753488 |
| 3F7 | JF753489 |
| 3F9 | JF753492 |
| 3F10 | JF753493 |
| 3F11 | JF753476 |
| 3G1 | JF753495 |
| 3G2 | JF753496 |
| 3G3 | JF753490 |
| 3G4 | JF753497 |
| 3G6 | JF753499 |
| 3G7 | JF753500 |
| 3G8 | JF753501 |
| 3G9 | JF753471 |
| 3G11 | JF753509 |
| 3H1 | JF753511 |
| 3H2 | JF753512 |
| 3H3 | JF753513 |
| 3H4 | JF753514 |
| 3H5 | JF753515 |
| 3H8 | JF753518 |
| 4A3 | JF776529 |
| 4A4 | JF776551 |
| 4A5 | JF776530 |
| 4A6 | JF776517 |
| 4A7 | JF776507 |
| 4A8 | JF776531 |
| 4A9 | JF776553 |
| 4A12 | JF776532 |
| 4B1 | JF776518 |
| 4B2 | JF776552 |
| 4B3 | JF776478 |
| 4B6 | JF776479 |
| 4B7 | JF776554 |
| 4B8 | JF776480 |
| 4B9 | JF776555 |
| 4B10 | JF776519 |
| 4B12 | JF776556 |
| 4C2 | JF776547 |
| 4C5 | JF776481 |
| 4C6 | JF776482 |
| 4C7 | JF776463 |
| 4C8 | JF776557 |
| 4C9 | JF776561 |
| 4C12 | JF776483 |
| 4D1 | JF776468 |
| 4D2 | JF776544 |
| 4D4 | JF776520 |
| 4D5 | JF776504 |
| 4D6 | JF776548 |
| 4D7 | JF776562 |
| 4D8 | JF776558 |
| 4D9 | JF776563 |
| 4D10 | JF776533 |
| 4D11 | JF776534 |
| 4D12 | JF776564 |
| 4 E1 | JF776502 |
| 4 E2 | JF776500 |
| 4 E3 | JF776535 |
| 4 E4 | JF776484 |
| 4 E5 | JF776476 |
| 4 E6 | JF776510 |
| 4 E7 | JF776485 |
| 4 E8 | JF776536 |
| 4 E9 | JF776565 |
| 4 E10 | JF776521 |
| 4 E11 | JF776522 |
| 4 E12 | JF776537 |
| 4F1 | JF776566 |
| 4F3 | JF776487 |
| 4F4 | JF776538 |
| 4F5 | JF776511 |
| 4F6 | JF776567 |
| 4F7 | JF776488 |
| 4F8 | JF776489 |
| 4F9 | JF776539 |
| 4F10 | JF776490 |
| 4F12 | JF776559 |
| 4G1 | JF776473 |
| 4G2 | JF776505 |
| 4G3 | JF776477 |
| 4G4 | JF776470 |
| 4G6 | JF776512 |
| 4G7 | JF776523 |
| 4G8 | JF776491 |
| 4G9 | JF776540 |
| 4G12 | JF776541 |
| 4H1 | JF776542 |
| 4H4 | JF776475 |
| 4H5 | JF776560 |
| 4H6 | JF776474 |
| 4H7 | JF776492 |
| 4H8 | JF776493 |
| 4H9 | JF776513 |
| 4H12 | JF776524 |
| 5A2 | JF776543 |
| 5A3 | JF776494 |
| 5A4 | JF776495 |
| 5A5 | JF776496 |
| 5A6 | JF776525 |
| 5A7 | JF776526 |
| 5A8 | JF776516 |
| 5A10 | JF776527 |
| 5A12 | JF776501 |
| 5B1 | JF776550 |
| 5B2 | JF776514 |
| 5B3 | JF776499 |
| 5B4 | JF776497 |
| 5B5 | JF776498 |
| 5B8 | JF776471 |
| 5B9 | JF776546 |
| 5B10 | JF776464 |
| 5B11 | JF776465 |
| 5B12 | JF776486 |
| 5C2 | JF776466 |
| 5C3 | JF776467 |
| 5C5 | JF776528 |
| 5C6 | JF776515 |
| 5C9 | JF776472 |
