# Supplementary figures and images for: Relevance of in vitro agar based screens to characterize the anti-fungal activities of bacterial endophyte communities
Source: BMC Microbiol. 2016 Jan 16;16:8. doi: 10.1186/s12866-016-0623-9 (PMC4715354; doi:10.1186/s12866-016-0623-9)

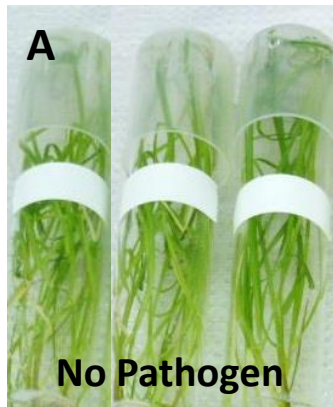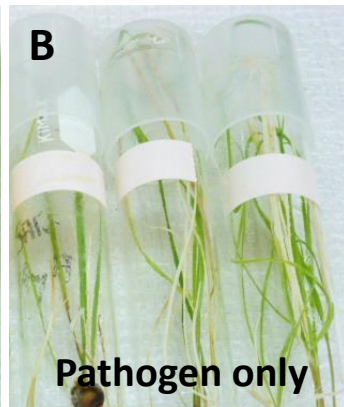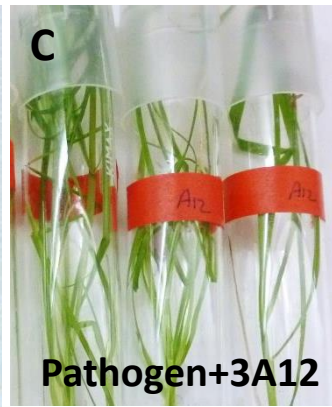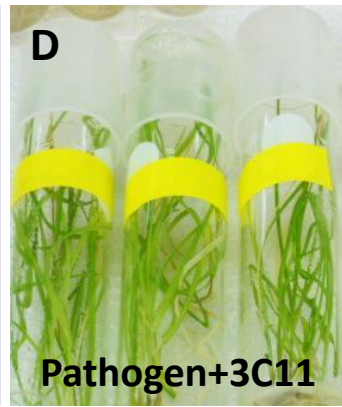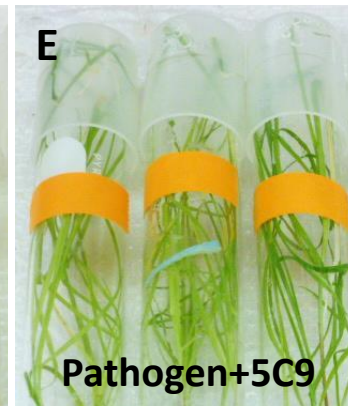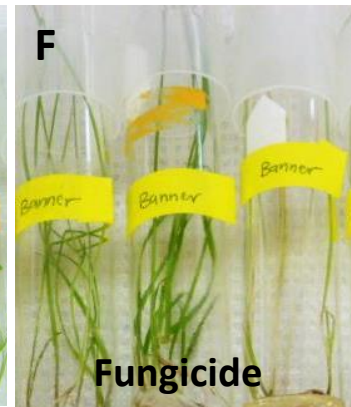

Supplement: Additional file 4: Figure S1. — In planta screening of maize endophytes for antifungal activity against S. homoeocarpa in annual ryegrass. Shown are tubes with annual ryegrass treated with (A) no fungal pathogen and no endophyte, (B) the pathogen but no endophyte, (C-E) the pathogen and successful anti-fungal endophytes, specifically (C) endophyte 3A12, (D) endophyte 3C11, and (E) endophyte 5C9, (F) fungicide treatment (Propiconazole). (PDF 198 kb) [file 12866_2016_623_MOESM4_ESM.pdf]
