# Supplementary material for: Relevance of in vitro agar based screens to characterize the anti-fungal activities of bacterial endophyte communities
Source: BMC Microbiol. 2016 Jan 16;16:8. doi: 10.1186/s12866-016-0623-9 (PMC4715354; doi:10.1186/s12866-016-0623-9)
Supplement: Additional file 5: Table S1. — The origins of the Zea genotypes used to isolate the endophytes noted in this study. (DOCX 16 kb) [file 12866_2016_623_MOESM5_ESM.docx]

Additional file 5: Table S1. The origins of the *Zea* genotypes used to isolate the endophytes noted in this study.

|  | ***Zea* seeds** | **Accession number** | **Collection site** |
| --- | --- | --- | --- |
| 1 | *Zea mays* ssp. *parviglumis* | CIMMYT:11355 | Arcelia Highway, Guerrero |
| 2 | *Zea mays* ssp. *mexicana* | CIMMYT:11386 | San Antonio Tlatenco, Mexico. |
| 3 | *Zea nicaraguensis* | CIMMYT: 11083 | Chinandega, Nicaragua |
| 4 | *Zea diploperennis* | CIMMYT:9476 | Las Joyas, Cuatitlan, Jalisco |
| 5 | Cristalino de Chihuahua | CIMMYT:6862 | CIMMYT, Mexico |
| 6 | Chapalote | CIMMYT:861 | CIMMYT, Mexico |
| 7 | Nal-Tel | CIMMYT:815 | CIMMYT, Mexico |
| 8 | Mixteco | CIMMYT:24143 | CIMMYT, Mexico |
| 9 | Bolita | CIMMYT:10503 | CIMMYT, Mexico |
| 10 | Jala | CIMMYT:2215 | CIMMYT, Mexico |
| 11 | Tuxpeno | CIMMYT:2536 | CIMMYT, Mexico |
| 12 | Gaspe Yellow Flint | NCPRIS: Pi 214279 | Castana, Iowa, USA |
| 13 | B73 | CIMMYT: 23811 | CIMMYT, Mexico |
| 14 | Pioneer 3751 | Pioneer 14498792 | Szarvas, Hungary |
